# Labeled breath tests in patients with NASH: Octanoate oxidation relates best to measures of glucose metabolism

**DOI:** 10.3389/fphys.2023.1172675

**Published:** 2023-04-21

**Authors:** Justine M. Mucinski, Alisha M. Perry, Talyia M. Fordham, Alberto Diaz-Arias, Jamal A. Ibdah, R. Scott Rector, Elizabeth J. Parks

**Affiliations:** ^1^ Department of Nutrition and Exercise Physiology, University of Missouri, Columbia, MO, United States; ^2^ Boyce & Bynum Pathology Professional Services, Columbia, MO, United States; ^3^ Department of Medicine, Division of Gastroenterology and Hepatology, University of Missouri School of Medicine, Columbia, MO, United States; ^4^ Research Service, Harry S. Truman Memorial Veterans Medical Center, Columbia, MO, United States; ^5^ NextGen Precision Health, University of Missouri, Columbia, MO, United States

**Keywords:** stable isotope, NASH, nonalcoholic steatohepatitis, glucose, insulin sensitivity, fatty acid oxidation

## Abstract

*In vivo* methods to estimate human liver mitochondrial activity are lacking and this project’s goal was to use a non-invasive breath test to quantify complete mitochondrial fat oxidation and determine how test results changed when liver disease state was altered over time. Patients with suspected non-alcoholic fatty liver disease (NAFLD; 9 men, 16 women, 47 ± 10 years, 113 ± 23 kg) underwent a diagnostic liver biopsy and liver tissue was histologically scored by a pathologist using the NAFLD activity score (0–8). To assess liver oxidation activity, a labeled medium chain fatty acid was consumed orally (23.4 mg ^13^C_4_-octanoate) and breath samples collected over 135 min. Total CO_2_ production rates were measured using breath ^13^CO_2_ analysis by isotope ratio mass spectrometry. Fasting endogenous glucose production (EGP) was measured using an IV infusion of ^13^C_6_-glucose. At baseline, subjects oxidized 23.4 ± 3.9% (14.9%–31.5%) of the octanoate dose and octanoate oxidation (OctOx) was negatively correlated with fasting plasma glucose (*r* = −0.474, *p* = 0.017) and EGP (*r* = −0.441, *p* = 0.028). Twenty-two subjects returned for repeat tests 10.2 ± 1.0 months later, following lifestyle treatment or standardized care. OctOx (% dose/kg) was significantly greater across all subjects (*p* = 0.044), negatively related to reductions in EGP (*r* = −0.401, *p* = 0.064), and tended to correlate with reduced fasting glucose (*r* = −0.371, *p* = 0.090). Subjects exhibited reductions in steatosis (*p* = 0.007) which tended to correlate with increased OctOx (% of dose/kg, *r* = −0.411, *p* = 0.058). Based on our findings, the use of an ^13^C-octanoate breath test may be an indicator of hepatic steatosis and glucose metabolism, but these relationships require verification through larger studies in NAFLD populations.

## 1 Introduction

Non-alcoholic fatty liver disease (NAFLD) is the most prevalent liver disease in the world (Younossi et al., 2017) and is characterized by an excess of liver fat, tissue injury, and insulin resistance (Korenblat et al., 2008; Hardy et al., 2016; Diehl and Day, 2017). The diagnosis of NAFLD and the more advanced form, non-alcoholic steatohepatitis (NASH), currently requires a liver biopsy to histologically grade the extent of steatosis, inflammation, and hepatocellular ballooning (Brunt et al., 2011; Pai et al., 2022). While multiple factors likely contribute to the pathogenesis of NASH, mitochondrial dysfunction is currently being investigated as a key component contributing to the progression of this disease (Sunny et al., 2011; Koliaki et al., 2015; Satapati et al., 2015). Impaired hepatic fatty acid oxidation (FAO), increased reactive oxygen species production, reduced respiration, and morphological mitochondrial changes in a setting of NAFLD and NASH have been demonstrated in cell culture (Stefanovic-Racic et al., 2008; da Silva et al., 2014), animal models (Ibdah et al., 2005; Zhang et al., 2007; Seo et al., 2008; Thyfault et al., 2009; Rector et al., 2010; Rector et al., 2013; Patterson et al., 2016; Morris et al., 2017), and human tissue (Sanyal et al., 2001; Pérez-Carreras et al., 2003; Kohjima et al., 2007; Nakamuta et al., 2008; Koliaki et al., 2015; Moore et al., 2022). Without obtaining additional tissue during a liver biopsy, the characterization of mitochondrial function is challenging, and the invasive nature of the biopsy renders monitoring disease progression difficult. Validated non-invasive methods to measure and track changes in hepatic mitochondrial function are needed.

Carbon-labeled breath tests using substrates that are fully oxidized to CO_2_ (e.g., ^13^C-methionine, ^13^C- ketoisocaproate, ^13^C-octanoate) have been used to measure hepatic mitochondrial activity (Miele et al., 2003; Portincasa et al., 2006; Banasch et al., 2011). For example, ^13^C- ketoisocaproate, a metabolite of the branch chain amino acid (BCAA) leucine, is reflective of mitochondrial activity (Portincasa et al., 2006) but may also be advantageous in a NAFLD population, as BCAA metabolism has been identified as a feature of NAFLD (Grenier-Larouche et al., 2022). Alternatively, labeled octanoate, a medium-chain fatty acid, has also been used in rodents (Shalev et al., 2010), and humans (Armuzzi et al., 2000; Miele et al., 2003; Schneider et al., 2005; Braun et al., 2006; Mawatari et al., 2009) to non-invasively estimate hepatic mitochondrial activity. Conflicting results have been reported in studies assessing the utility of the labeled octanoate breath test in subjects with NAFLD or NASH (Miele et al., 2003; Schneider et al., 2005; Braun et al., 2006; Mawatari et al., 2009). For example, patients with NASH had elevated ^13^CO_2_ recovery when compared to healthy controls matched for age, gender, and BMI (Miele et al., 2003), while two other studies found no difference in octanoate oxidation (OctOx) between NASH and healthy subjects (Schneider et al., 2005; Braun et al., 2006). Yet another study found reduced OctOx in NASH compared to NAFLD (Braun et al., 2006). Importantly, no investigations have compared OctOx in biopsy-proven NASH patients with measures of liver glucose metabolism, nor have the effects of a lifestyle intervention in NASH patients on OctOx been evaluated.

The purpose of the current study was to determine how hepatic mitochondrial activity, measured via ^13^C-OctOx breath test, was related to indicators of liver health in individuals with biopsy-proven NASH. Additionally, in a subset of subjects, repeat OctOx breath tests were used to understand how oxidation changed in patients whose liver disease improved, stayed the same, or worsened over 10 months. We hypothesized that OctOx would be negatively related to characteristics of NASH while improvements in liver health would result in increased OctOx.

## 2 Methods

### 2.1 Protocol

The study was approved by the University of Missouri (MU) Health Sciences Institutional Review Board (Protocol # 2008258) and registered under ClinicalTrials.gov #NCT03151798. All subjects provided written informed consent and the study was conducted according to the principles expressed in the Declaration of Helsinki. Subjects (n = 25) with NASH were recruited following a diagnostic liver biopsy for histologic grading of liver disease. Liver biopsy tissues were reviewed by a single pathologist and graded for NAFLD activity score via the Brunt criteria (Brunt et al., 2011; Pai et al., 2022), which is made up of three components: steatosis, lobular inflammation, and hepatocellular ballooning. Additional eligibility criteria included being sedentary (<60 min/week of structured physical activity), 22–65 years of age, consuming less than two standard alcoholic drinks per day (<20 g/d), and possessing characteristics of metabolic syndrome (Grundy et al., 2004). Individuals with acute disease, advanced cardiac or renal disease, anticoagulation therapy, severe comorbid conditions limiting life expectancy <1 year, hepatitis-causing illnesses (hepatitis B and/or C viruses, autoimmune hepatitis, hemochromatosis, celiac disease, Wilson’s disease, alpha-1-antitrypsin deficiency, medication-induced hepatitis, or any other clinical or biochemical evidence of decompensated liver disease), steroid or drug use known to cause NAFLD, or pregnant women were excluded.

**FIGURE 1:**
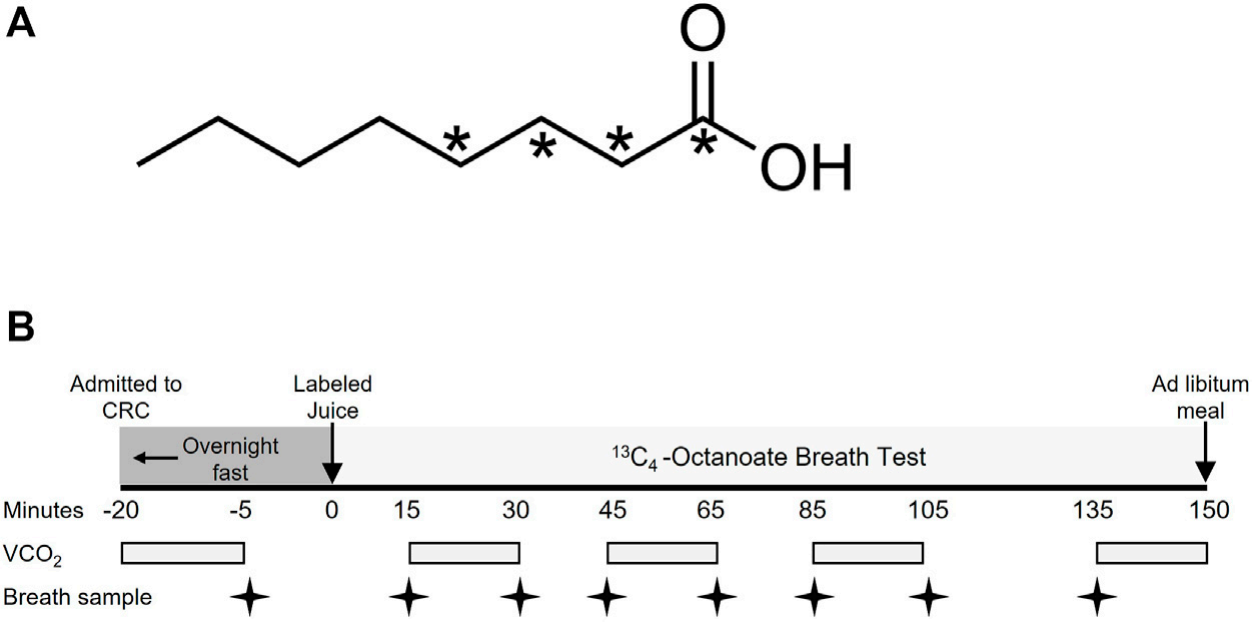
Molecular structure of labeled octanoate and study design of the breath test. **(A)** indicates the position of the ^13^C within the molecule. **(B)** Subjects completed a 12-h overnight fast prior to the breath test. VCO_2_, volume of carbon dioxide production in L/min.

In Part 1 of this research, within 3.0 ± 1.8 months of the liver biopsy, a fatty acid breath test was performed in which 1,2,3,4 ^13^C-octanoic acid (Figure 1A; isotopic purity: 99%, Cambridge Isotope Laboratories, Andover, MA) was delivered orally in orange juice, as described below. Because acute changes in energy intake and body weight may influence liver metabolism (Schwarz et al., 1995), prior to the test, each subject completed a 3-day food record and food preference surveys which were used to prepare a controlled 3-day, pre-study diet. The prepared diets were similar in composition and energy content to each subjects’ habitual food preferences but were provided by the research team to support subject body weight maintenance over 3 days. The breath test was performed on day 4, following a 12 h fast. Approximately 2 weeks after the breath test, subjects were admitted to the Clinical Research Center (CRC) at the MU Hospital and underwent a stable-isotope labeled glucose infusion to determine endogenous glucose production (EGP) in the fasting state, as previously described (Ramos-Roman et al., 2020).

In Part 2, data were analyzed from twenty-two subjects who returned after 10.2 ± 1.0 months for follow-up tests. These subjects were those involved in a project to receive either standard of care (n = 7) or a supervised lifestyle intervention (n = 15). The subjects not retained for follow-up (n = 3) dropped out, did not complete the OctOx test, or were removed from the study for non-compliance (i.e., multiple missed appointments). Subjects receiving standard care met with a registered dietitian once at the beginning of the study to discuss lifestyle changes that could improve their liver health (after their first liver biopsy); they also received all food for 2 weeks to aid them in learning about healthful eating patterns. Any additional instruction was provided by their physician or clinical staff independently of the research program. The subjects in the lifestyle program regularly met with the study dietitian for nutritional counseling (2–4x/month) and an exercise physiologist for supervised exercise (see Supplementary Section). At the end of the program, each subject completed a follow-up OctOx breath test, measurements of EGP, and underwent a follow-up liver biopsy to document changes in liver health. The pathologist grading liver biopsies was blinded to subject group assignments and visit.

### 2.2 Octanoate oxidation breath test

The protocol for the OctOx test performed at baseline and follow-up is shown in Figure 1B. After admission to the CRC, fasting CO_2_ production rate was measured at approximately 0700 (-20 min), using a metabolic cart (Parvomedics, Salt Lake City, UT). An unlabeled breath sample was collected into an Exetainer® evacuated breath vial (Labco, Ceredigion, United Kingdom) using a disposable plastic straw. Each subject then consumed 23.4 mg of ^13^C_4_-octanoate (25 μL, Figure 1A) mixed thoroughly into orange juice (Minute Maid®; Sugar Land, TX) to act as a carrier of the isotope and to mimic energy intake occurring at breakfast. Fed-state CO_2_ production rates were measured four times over 135 min, each time for 15 min. Breath samples were collected intermittently throughout the test at 15, 30, 45, 65, 85, 105, and 135 min (Figure 1B). Because previous studies have monitored oxidation 120 min after the consumption of the label (Miele et al., 2003; Schneider et al., 2005), we chose to collect data for a slightly longer period to capture a greater timeframe of oxidation. The amount of ^13^CO_2_ in the breath samples was measured by Metabolic Solutions (Nashua, NH) with a Sercon ABCA2 isotope ratio mass spectrometer (IR-MS, Sercon, Ltd., Crewe, United Kingdom) and OctOx was calculated as thoroughly described in the Supplementary Methods. In brief, the product of ^13^C atom excess and CO_2_ production rates (metabolic cart) were used in Eq. 1 to calculate total OctOx throughout the test. Following the test, subjects ate *ad libitum* and were discharged from the CRC. Within 2 weeks of the breath test, subjects returned to undergo an isotope infusion of glucose to quantitate fasting EGP. Two anterograde intravenous lines were placed—one for isotope administration in the antecubital region and one for blood draws from a hand IV, which was kept warm using a heated hand box (Abumrad et al., 1981). A primed-continuous infusion of [U-^13^C_6_]-glucose (22 μmol/kg over 1 minute, followed by 0.2 μmol/kg/min) was administered. Plasma glucose enrichments were measured by gas chromatography/mass spectrometry (Wolfe, 1992) and EGP was calculated according to the equations of Steele (Steele et al., 1956).

$$\left(\frac{\left(\frac{\left(VCO_{2}*Atome\;excess\right)}{Bicarbonate\;factor\;\left(0.81\right)}\right)}{4}\right)*148.18\left(\frac{g}{mol}\right)$$ (1)

### 2.3 Statistical analysis

The homeostatic model assessment for insulin resistance (HOMA-IR) was calculated by the product of fasting insulin (µIU/mL) and glucose (mg/dL) divided by the constant 405 (Matthews et al., 1985) and the model for end-stage liver disease (MELD) was calculated as previously described (Singal and Kamath, 2013). Calculations were performed using Microsoft Excel 2013 and correlation analyses (Pearson for parametric, Spearman for non-parametric—continuous versus ranked variables) using StatView®, 5.0.1 software (2008). In Part 2 of this study, paired, one-tailed *t*-tests were used to compare baseline to follow-up characteristics, based upon the a prior hypothesis that increased OctOx would be related to improvements in markers of liver health. Comparison of outcomes between the groups was performed using repeated measures analysis of variance and post-hoc tests corrected with the Bonferroni adjustment (Table 1). An alpha level of ≤ 0.05 was considered significant while ≤ 0.10 was considered a trend. Data are presented as mean ± SD for static variables (i.e., body weight, age) and as mean ± SEM for values measured over time.

**TABLE 1:** Subject characteristics.

	Part 1	Part 2				
	All subjects	Standard of care		Treatment		ANOVA
Characteristic	Baseline (n = 25)	Baseline (n = 7)	Follow-Up (n = 7)	Baseline (n = 15)	Follow-Up (n = 15)	*p-*value
Sex (m/f)	9/16	2/5	2/5	7/8	7/8	
Age (y)	47 ± 10	46 ± 11	47 ± 11	46 ± 10	47 ± 9	0.584^╪^
Body Weight (kg)	113 ± 23	107 ± 25	105 ± 21	119 ± 22	111 ± 19	0.141^╪^
NAFLD activity score (0–8)	5.5 ± 1.1	5.6 ± 1.0	5.9 ± 0.7	5.5 ± 1.2*	3.1 ± 1.9	0.002^#, ╪^
Steatosis (0–3)	2.3 ± 0.6	2.7 ± 0.5	2.6 ± 0.5	2.2 ± 0.6	1.6 ± 1.0	0.220^#, ╪^
Inflammation (0–3)	1.6 ± 0.6	1.6 ± 0.5	1.9 ± 0.4	1.7 ± 0.6*	1.0 ± 0.8	0.003
Ballooning (0–2)	1.5 ± 0.6	1.3 ± 0.8	1.4 ± 0.5	1.6 ± 0.5*	0.5 ± 1.7	<0.001^╪^
Fibrosis Score (0–4)	1.9 ± 1.5	1.7 ± 1.7	2.3 ± 1.6	2.0 ± 1.3	1.5 ± 1.6	0.045
AST (U/L)	51 ± 37	50 ± 33	33 ± 17	57 ± 40	26 ± 9	0.390^╪^
ALT (U/L)	62 ± 39	75 ± 52	39 ± 13	61 ± 35	30 ± 13	0.778^╪^
INR^1^	1.0 ± 0.1	0.9 ± 0.1	1.0 ± 0.1	1.0 ± 0.1	1.0 ± 0.1	0.951
Plasma albumin (g/dL)	4.4 ± 0.4	4.2 ± 0.4	4.4 ± 0.4	4.4 ± 0.4	4.6 ± 0.4	0.360
Platelet count (K/mm^2^)	240 ± 71	231 ± 57	222 ± 36	250 ± 77	258 ± 84	0.698^╪^
MELD score^1^	6.0 ± 1.5	5.6 ± 0.6	6.1 ± 1.0	6.2 ± 1.9	6.5 ± 1.7	0.977
Fasting glucose (mg/dL)	133 ± 46	143 ± 48	131 ± 42	128 ± 50	115 ± 37	0.965
HOMA-IR	6.0 ± 4.5	8.0 ± 6.3	5.1 ± 1.2	5.4 ± 3.6	3.4 ± 1.6	0.670^╪^
Fasting EGP (µmol/FFM/min)	21.8 ± 3.4	22.5 ± 4.2	23.8 ± 5.9	21.2 ± 3.2	20.6 ± 3.0	0.418
Octanoate oxidized (% dose)	23.4 ± 3.9	23.3 ± 2.3	23.5 ± 4.2	23.8 ± 4.2	24.2 ± 4.7	0.892
Octanoate oxidized (% dose/kg)	0.22 ± 0.06	0.23 ± 0.05	0.24 ± 0.08	0.21 ± 0.05	0.23 ± 0.07	0.811

All values are mean ± SD. Part 1 included 25 subjects studied at weight stability, while in Part 2, a subset of the Part 1 subjects (n = 22) completed repeat tests ∼10 months later after they were enrolled in either lifestyle treatment (n = 15) or standard of care (n = 7). *p*–values represent repeated measures ANOVA to test group by time effects in Part 2. Significant (*p* < 0.05) main effects of group are denoted with a ^#^ while a main effect of time is represented by ^╪^. Post-hoc analyses with Bonferroni adjustments were run and significance within a group (e.g., values in treated subjects, baseline versus follow-up) is represented by an asterisk (*p* < 0.05). NAFLD activity score was derived from histological evaluation of the liver biopsy.

^1^ Baseline values available for 13 treatment subjects and 6 for standard of care subjects; MELD was calculated according to (Singal and Kamath, 2013): (9.57*loge ([Creatinine]))+(3.78*loge([Bilirubin]))+(11.2*loge ([INR]))+6.43.

Abbreviations: ALT, alanine aminotransferase; AST, aspartate aminotransferase; EGP, endogenous glucose production; FFM, fat free mass; HOMA-IR, homeostatic model assessment for insulin resistance; INR, international normalized ratio; MELD, model for end-stage liver disease; NAFLD, non-alcoholic fatty liver disease.

## 3 Results

In Part 1 of this project, baseline studies were performed in 25 subjects diagnosed with NASH, and in Part 2, a subset of the subjects (n = 22) were restudied 10 months later. In the intervening time, some of the participants had improved their liver health and others had not. Subject characteristics are shown in Table 1. All subjects were recruited based on a biopsy-confirmed diagnosis of NASH (NAFLD activity score ≥ 4/8) which is the sum composite of scores for steatosis (0–3), inflammation (0–3), and hepatocellular ballooning (0–2). Additionally, subjects demonstrated multiple characteristics of liver disease including fibrosis (measured histologically), elevated serum aspartate transaminase (AST), alanine transaminase (ALT), and HOMA-IR. The majority of the subjects (72%) had type 2 diabetes (T2D) and their medication use is reported in Supplementary Table S1.

**FIGURE 2:**
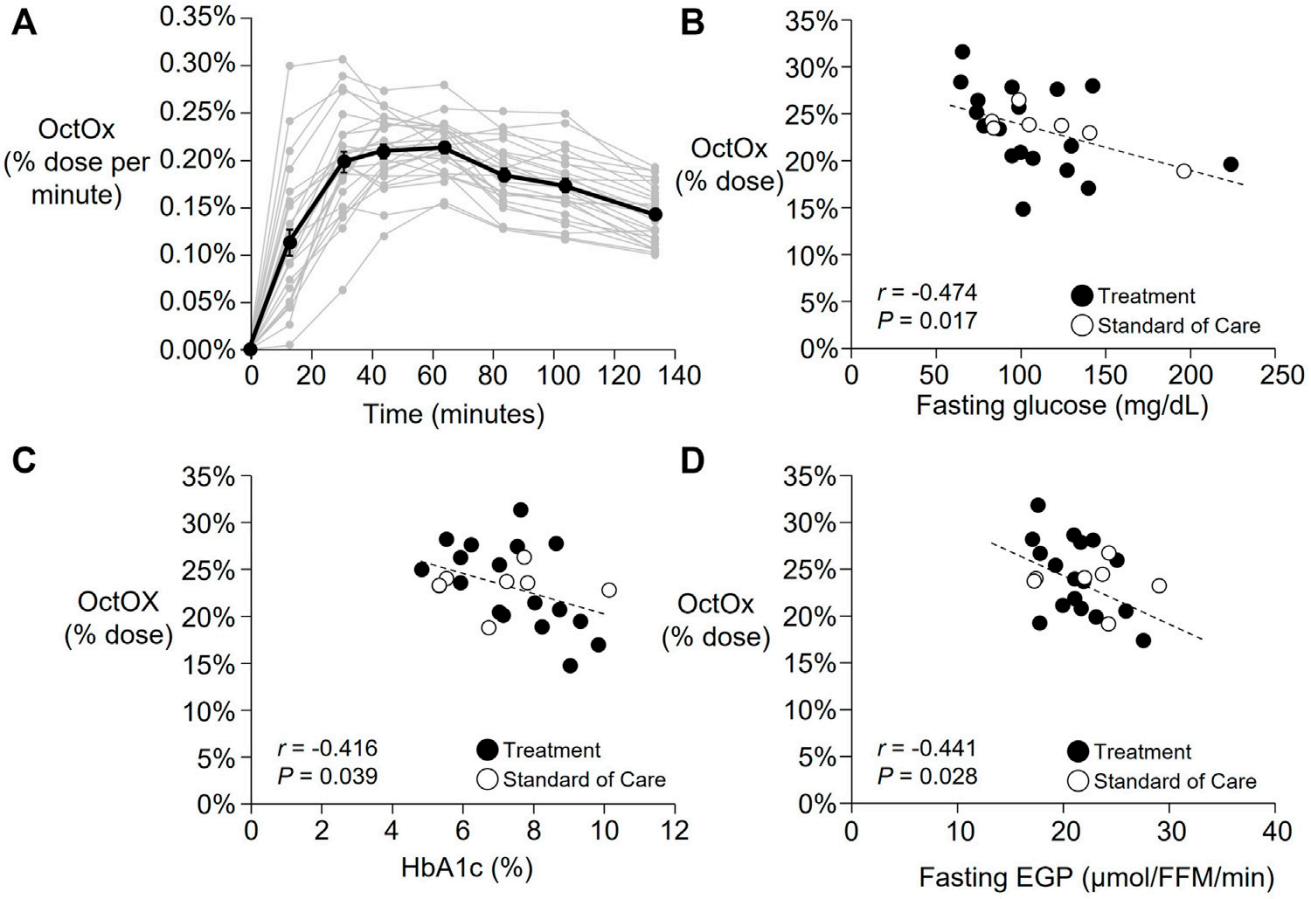
Part 1: Baseline time courses of the percentage of the octanoate dose oxidized (OctOx) per minute and correlations with baseline markers of glucose metabolism. **(A)** Percentage of the octanoate dose oxidized per minute are shown for all baseline tests (n = 25). Mean data shown in black line with individual subject data are shown in grey. (**B–D)**: Pearson’s correlations between baseline percentage oxidation of the oral octanoate dose over 135 min (n = 25) and **(B)** fasting plasma glucose concentration, **(C)** HbA1c, and **(D)** fasting endogenous glucose production (EGP) relative to fat free mass (FFM). While all subjects were analyzed together at baseline, the subjects that were randomized to standard of care are shown in unfilled circles (n = 7) and those in treatment in filled circles (n = 18). Three subjects initially randomized to the treatment group did not complete follow-up testing.

As shown in Figure 2A, a wide variability in OctOx rates were observed across subjects throughout the breath test. Average peak oxidation occurred at 53 ± 20 min following ingestion (range: 23–104 min). Total octanoate oxidized within the 135 min was 23.4 ± 3.9% of the dose (Table 1, range: 14.9%–31.5%), or in units accounting for body weight, 0.22 ± 0.06%/kg (range: 0.13%–0.33%/kg). No association was found between baseline body weight, lean mass, NAFLD activity score, liver fibrosis, or plasma liver enzymes and % dose oxidized. However, OctOx was significantly associated with multiple indicators of glucose metabolism. The higher the OctOx the lower the fasting plasma glucose concentration (Figure 2B), glycosylated hemoglobin (HbA1c, Figure 2C), and fasting EGP (Figure 2D).

**FIGURE 3:**
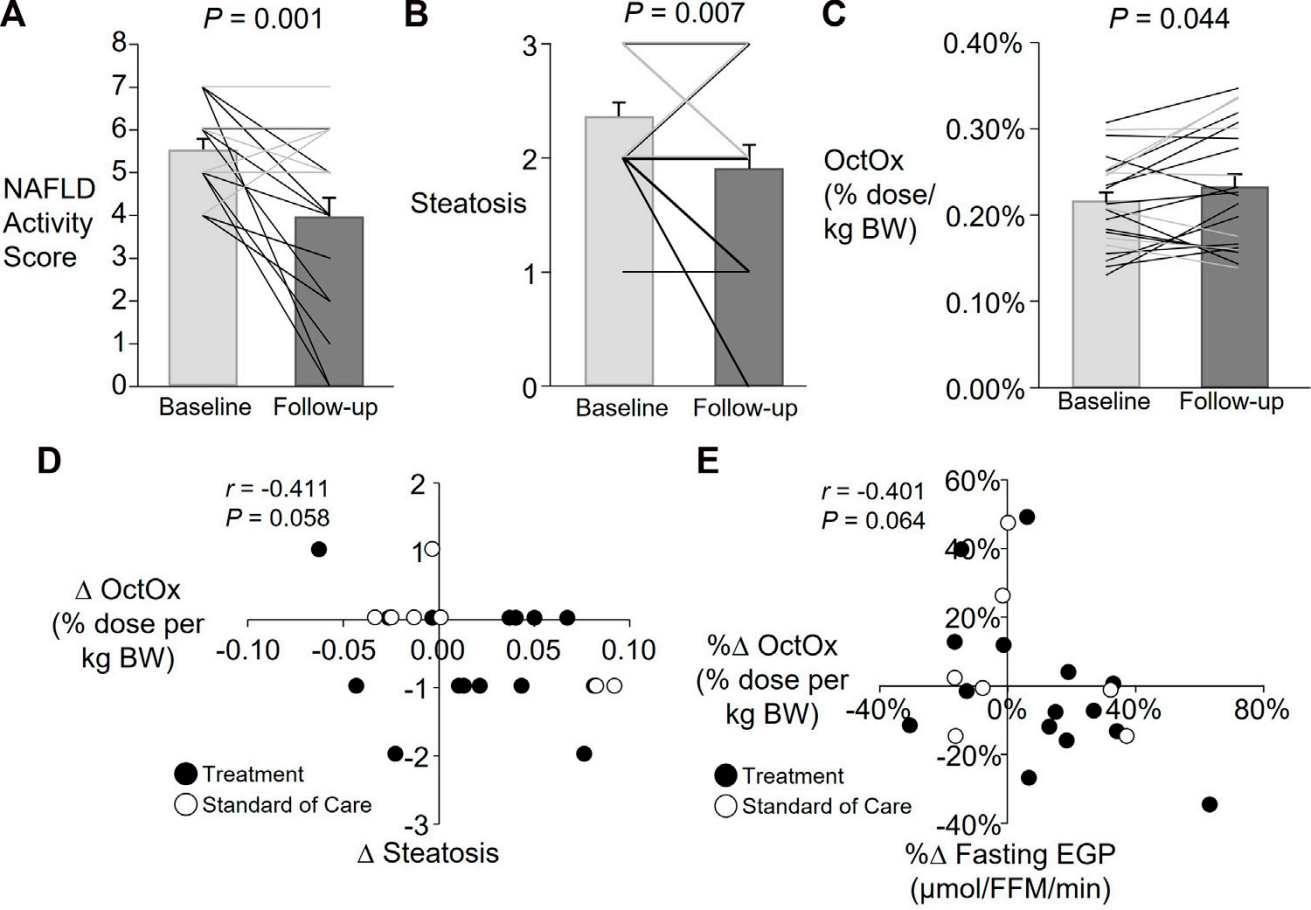
Part 2: Average and individual changes in NAFLD activity score, steatosis, and percent (%) octanoate dose oxidized (OctOx) and relationships between change in OctOx, steatosis, and glucose production. Data are presented as mean ± SEM; Lifestyle treatment: n = 15, solid black lines and black-filled circles; Standard care: n = 7, solid grey lines and white-filled circles. Paired, one-tailed *t*-test for baseline to follow-up comparisons in all subjects. Note: Many individual subject lines overlap and individual data are presented in Supplementary Figure S1. **(A)** Changes in total NAFLD activity score. **(B)** Changes in the steatosis component of the NAFLD activity score. **(C)** Changes in OctOx, expressed as a percentage of the total dose oxidized per kg body weight over 135 min. **(D)** Spearman’s non-parametric correlation between absolute changes (∆) in steatosis and percent (%) dose OctOx per kg body weight. **(E)** Pearson’s correlation between percent changes in fasting EGP (relative to fat free mass, FFM) and percent dose OctOx per kg body weight.

Presented in Figure 3 are data from all subjects participating in Part 2 (denoted by bar graphs) and also individual data with lifestyle treatment subjects and standard of care subject subjects. Supplementary Figure S1 presents the same data for each individual which shows that over 10 months, different subjects in the lifestyle treatment arm and the standard care arm demonstrated both improved and worsening measures of health, although those in the lifestyle arm improved their NAFLD activity score more (Figure 3A, note black lines on graph). On average, subjects in Part 2 lost 6 ± 1% of their body weight (average ± SD for standard of care and treatment subjects combined), serum AST and ALT were reduced 67 ± 20% and 38 ± 7% respectively, and HOMA-IR decreased by 14 ± 12% at follow-up. Group differences are shown in Table 1, with significant group by time effects found for the liver NAFLD activity score (*p* = 0.002), inflammation *p* = 0.003), ballooning (*p* < 0.001), and fibrosis score (*p* = 0.045). Fasting EGP (relative to fat free mass, FFM) was not different at follow-up and no group by time effect was detected (Table 1). When analyzed as a group, total NAFLD activity score was significantly reduced (*p* = 0.001, Figure 3A), which was driven by subjects in the lifestyle arm. Steatosis was also reduced over time (*p* = 0.007, Figure 3B). Some subjects exhibited robust reductions in NAFLD activity score and steatosis while others had minimal changes or even increased (Figures 3A,B and Supplementary Figure S1A–B). Across all subjects, liver fibrosis did not change significantly (*p* = 0.246, data not shown).

With regard to the OctOx, when all subjects were analyzed together, a significant increase in the total percentage of dose oxidized per kg body weight was found (Figure 3C, *p* = 0.044). No relationship was evident between changes in OctOx and NAFLD activity score. However, as shown in Figure 3D, over time, as steatosis was reduced, OctOx tended to increase. Neither lobular inflammation and hepatocellular ballooning (the other components of the NAFLD activity score), nor fibrosis were related to changes in OctOx. However, as plasma ALT fell, absolute change in OctOx tended to increase (data not shown; *r* = −0.405, *p* = 0.068). Finally, the percent change in relative OctOx tended to correlate with absolute changes in fasting glucose (data not shown, *r* = −0.371, *p* = 0.090) and improvements in OctOx and EGP tended to be related; those subjects that reduced their fasting glucose production rates, exhibited greater oxidation of octanoate during the test (Figure 3E).

## 4 Discussion

The present study utilized an *in-vivo*, isotope-labeled breath test in subjects with biopsy-proven NASH to determine the relationships between medium chain fatty acid (octanoate) oxidation and measures of liver health. A subset of the subjects underwent a second breath test and liver biopsy after 10 months to show, for the first time, a significant relationship between the changes in OctOx and fasting glucose production. OctOx, which was greater at follow-up, tended to increase when reductions in steatosis and fasting EGP occurred over time. These findings were supported by relationships between changes in OctOx and ALT, a plasma marker of liver injury, and fasting glucose concentrations. Overall, increased OctOx may be a non-invasive indicator of improved liver health and should be further tested as a predictor of glucose production and liver fat in subjects with NASH.

### 4.1 Liver health and mitochondrial function: Insight from previous breath tests

Although liver biopsies used to grade disease severity provide information on a small segment of the liver, no non-invasive test to date, whether serum-based or imaging, has been able to characterize disease severity as precisely as the liver biopsy (Piazzolla and Mangia, 2020). The use of breath tests to measure hepatic function began nearly 50 years ago when Hepner and Vesell quantified hepatic drug metabolism in patients with portal cirrhosis using radiolabeled aminopyrine (Hepner and Vesell, 1974). Since then, numerous breath tests have been developed to measure liver function (Armuzzi et al., 2000; Armuzzi et al., 2002; Miele et al., 2003; Portincasa et al., 2006; Banasch et al., 2011; Bonfrate et al., 2015), as comprehensively reviewed elsewhere (Di Ciaula et al., 2021). For a medium-chain fatty acid like octanoate, several independent studies suggest that when it is given orally, its oxidation is specific to the liver (Bloom et al., 1951; Papamandjaris et al., 1998; DeLany et al., 2000). When consumed, octanoic acid is absorbed into enterocytes and transported to the liver via the portal vein (Bloom et al., 1951). Once in the liver, octanoate bypasses the carnitine transport system (Fritz, 1963), diffusing directly into the mitochondria and exhibiting efficient oxidation (Bremer, 1980). Acetyl coenzyme A is then produced during β-oxidation (Bach and Babayan, 1982; Wolfe and Jahoor, 1990; Guillot et al., 1993; DeLany et al., 2000) and flows through the tricarboxylic acid (TCA) cycle producing CO_2_ which is expelled in the breath. In the present study, the carbon from ^13^C-labeled octanoate appeared in human breath CO_2_ within 15 min and peaked, on average, within 60 min (Figure 2A). We also analyzed by GC/MS, plasma samples drawn from the periphery via the antecubital vein blood draw and did not detect any labeled octanoate in those samples (data not shown) during the 135 min of data collection. The sum of these data support that octanoate is cleared first past by the liver.

Part 1 of this project was an observational study to determine the relationships between OctOx and the metabolic phenotype of patients with NAFLD. Recent work from our group has shown downregulation of *in vitro* FAO in liver tissue collected from individuals with more advanced NAFLD and NASH (Moore et al., 2022). In light of these findings, we were surprised to find no association between NAFLD activity score and OctOx. On the other hand, multiple independent characteristics of glucose metabolism were related to OctOx (Figures 2B–D) demonstrating that those subjects who exhibited the greatest metabolic dysfunction as evidenced by elevated glucose levels, HbA1c, and EGP, were also those who oxidized the least octanoate during the test. Braun et al. (Braun et al., 2006) showed that subjects with NASH exhibited lower OctOx compared to subjects with lesser levels of liver disease (i.e., steatosis only). Similarly, Banasch et al. showed that worsening of liver disease was associated with reduced oxidation, indicative of mitochondrial dysfunction, when using a ^13^C-methionine breath test (Banasch et al., 2011). Additionally, a ^13^C- ketoisocaproate test performed by Portincasa et al. demonstrated reduced mitochondrial decarboxylation, a reflection of mitochondrial activity of the cytochrome P450 system, in NASH compared to healthy subjects or those with steatosis alone (Portincasa et al., 2006). However, not all studies have agreed with these findings. Using similar methods as the current study, no differences in OctOx were reported in patients with NASH vs. healthy controls (Schneider et al., 2005) and one other study found *greater* OctOx in subjects with NASH compared to healthy controls (Miele et al., 2003). Although the current study did not include a healthy group for comparisons, the goal of Part 2 of this project was to measure changes in OctOx over time, when liver disease severity may be changing.

### 4.2 Improvements in NASH and hepatic FAO

Previous studies in humans with NASH have shown that lifestyle treatment can significantly reduce disease severity (Vilar-Gomez et al., 2015). We also observed reductions in NAFLD activity score (-44%) and steatosis (-28%) at follow-up in subjects who had been treated with diet and exercise, suggesting that this treatment may have increased mitochondrial oxidative capacity and reduced cellular inflammation thereby improving the ability of the mitochondria to burn fatty acids. Indeed, we did observe a significant increase in relative OctOx (Figure 3C) where the change tended to be associated with reduced liver steatosis (Figure 3D), however we found no association between hepatic inflammation or fibrosis and OctOx. An important caveat of comparing our results to previous studies using long chain fatty acids (LCFA) as metabolic probes, is that octanoate does not require the carnitine palmitoyl transferase (CPT) shuttle to transfer the fatty acid into the mitochondria (Lira et al., 2012). As a result, the octanoate breath test provides a readout of the latter stages of FAO including β-oxidation and TCA cycle activity. We found that increases in OctOx tended to correlate with reductions in steatosis (indicative of liver triacylglycerols containing primarily LCFA), which may suggest that increased octanoate metabolism is indicative of the global oxidation of liver fat. Future studies should assess if increased FAO is also reflective of decreases in hepatic lipogenesis, through relief of inhibition by malonyl CoA (Randle et al., 1963), which may also contribute to changes in steatosis levels, as were observed here. Despite the lack of association with markers of advanced NASH (e.g., fibrosis, inflammation), OctOx may be a marker of glucose metabolism, a key factor in the progression of NAFLD and other metabolic diseases.

### 4.3 Hepatic mitochondrial activity and glucose production

A key event in the pathogenesis of NAFLD is increased substrate burden (Tamura et al., 2005; Westerbacka et al., 2005; Cusi, 2012) which overwhelms the liver’s capacity to oxidize, store, and secrete metabolites, ultimately leading to the accrual of liver fat (Sanyal et al., 2001; Iozzo et al., 2004; Fabbrini et al., 2008). This event occurs concurrently with a decline in mitochondrial function (Koliaki et al., 2015; Moore et al., 2022) and significant structural defects in hepatic mitochondria from NASH patients (Sanyal et al., 2001). Simultaneously, in states of overnutrition, surplus nutrient burden induces insulin resistance (McGarry, 1992; Unger, 1995). As a result, the liver’s ability to suppress gluconeogenesis is blunted, resulting in excess glucose production, which is the primary driver of elevated plasma glucose concentrations in the fasting state (Korenblat et al., 2008).

In Part 1 of the project, lower levels of OctOx were related to multiple independent indicators of hepatic insulin resistance—i.e., those participants with the lowest OctOx exhibited greater fasting EGP, fasting plasma glucose concentrations, and the highest HbA1c (Figure 2). In Part 2, follow-up studies in a subset of the participants demonstrated that OctOx was significantly increased (Figure 3C) and tended to correlate to reductions in EGP (Figure 3E). When fasting ALT, glucose, and steatosis fell, OctOx tended to increase. These data suggest that an early event in improving liver health is an increase in FAO, whether this precedes or follows reductions in EGP requires further investigation.

### 4.4 Strengths and limitations

The primary limitation of this study relates to the nature of metabolic protocols in which multiple measurements are collected over time (e.g., biopsies, OctOx, isotope infusions, and mass spectrometry), which limits the sample size. Non-etheless, the relationships observed were strengthened by the study’s repeated-measures design and the wide range of values for OctOx and other variables, which highlight the importance of examining individual responses. The protocol controlled for food intake and physical activity in the days leading up to the test because acute effects of physical activity during the days preceding data collection may impact both hepatic FAO (Gorski et al., 1990; De Souza et al., 2010; Fuller et al., 2019) and insulin sensitivity (Devlin et al., 1987; Kirwan et al., 2009; De Souza et al., 2010). A second limitation relates to the fact that all subjects had advanced NASH (NAFLD activity scores ranging from 4 to 7). To determine whether OctOx level is lowered consistently at every increase in NAFLD activity score (from 0 to 8), future studies should include individuals with lower levels of liver disease at baseline, and even patients who are healthy, if liver tissue is available for histology. Third, multiple factors may have impacted an individual’s breath test response including subject sex (higher ^13^CO_2_ recovery was found in one study in women compared to men (Schneider et al., 2005)), portal blood flow, or gastric emptying (Mucinski et al., 2020; Jacome-Sosa et al., 2021). Portal blood flow is reduced with increasing steatosis and fibrosis (Kobayashi et al., 2009; Shigefuku et al., 2012) and may have impacted the delivery of octanoate to the liver. However, we found no differences in time to peak after significant liver health improvements which we would have expected if blood flow was compromised, thus we do not believe changes in portal flow significantly affected our results. Additionally, we chose to use a liquid, non-fat-containing vehicle (orange juice) to reduce any delay associated with solid food meals or fat-induced delays in gastric emptying. We found no correlation between the time to peak oxidation and the total percent OctOx occurring during the test, suggesting gastric emptying also did not influence the results. Lastly, instead of separately measuring the extent of CO_2_ trapping in the bicarbonate pool for each subject, we used an established correction factor to estimate this outcome (van Hall, 1999). The present subjects’ body weights and ages were similar to the original cohort used to establish the correction factor and we chose to use this factor to reduce subject burden. Nevertheless, future studies should assess individual bicarbonate correction factors to determine if they impact the results or reproducibility of the data. A key strength lies within the non-invasive nature of the OctOx breath test. Currently, methods for measurement of mitochondrial function are highly invasive (liver biopsies for *in vitro* FAO assays using labeled palmitate or fatty-acid stimulated oxygen consumption) although they yield results directly related to FAO whereas the OctOx method more generally represents intermediary metabolism.

## 5 Conclusions

In summary, the use of a non-invasive, medium-chain FAO breath test demonstrated that the oxidation of orally-administered octanoate was associated with hepatic glucose tolerance and changes in liver fat and glucose production in subjects with NASH. While more studies are needed to validate these associations across a range of liver disease, octanoate breath tests may be a simple method for simultaneously capturing progression or changes in liver fat content and hepatic EGP in subjects with biopsy-confirmed NASH.

## Data Availability

The raw data supporting the conclusions of this article will be made available by the authors, without undue reservation.

## Abbreviations

ALT, alanine aminotransferase; AST, aspartate aminotransferase; CRC, clinical research center; CPT, carnitine palmitoyl transferase; EGP, endogenous glucose production; FFM, fat free mass; HIIT, high intensity interval training; HOMA-IR, homeostatic model assessment for insulin resistance; NAFLD, nonalcoholic fatty liver disease; NASH, nonalcoholic steatohepatitis; OctOx, octanoate oxidized; TCA, tricarboxylic acid; T2D, type 2 diabetes.
