# Supplementary material for: Labeled breath tests in patients with NASH: Octanoate oxidation relates best to measures of glucose metabolism
Source: Front Physiol. 2023 Apr 21;14:1172675. doi: 10.3389/fphys.2023.1172675 (PMC10160408; doi:10.3389/fphys.2023.1172675)
Supplement: Supplementary file 1 [file DataSheet1.PDF]

Supplementary Material

Labeled breath tests in patients with NASH: Octanoate oxidation relates best to measures of glucose metabolism

Justine M. Mucinski[1], Alisha M. Perry[1], Talyia M. Fordham[1], Alberto Diaz-Arias[2], Jamal A. Ibdah[3,4], R. Scott Rector[1,3,4,5], Elizabeth J. Parks[1,3,5]*

[1] Department of Nutrition and Exercise Physiology, University of Missouri, Columbia, MO, USA
[2] Boyce & Bynum Pathology Professional Services, Columbia, MO, USA
[3] Department of Medicine, Division of Gastroenterology and Hepatology, University of Missouri School of Medicine, Columbia, MO, USA
[4] Research Service, Harry S Truman Memorial Veterans Medical Center, Columbia, MO, USA
[5] NextGen Precision Health, University of Missouri, Columbia, MO, USA

*** Correspondence:**
Elizabeth J. Parks, PhD
One Hospital Drive School of Medicine, NW 406
University of Missouri
Columbia, Missouri 65212
Email: parksej@missouri.edu

Octanoate breath test: $^{13}C_4$-Octanoate (23.4 mg, Cambridge Isotope Laboratories, Andover, MA, product # CLM-2721) was dissolved into orange juice (Minute Maid 100% orange juice). The volume of juice provided energy, equivalent to 10% of each subject's daily energy requirement calculated using the Harris Benedit equation (Harris and Benedict, 1918) and including an activity factor. The juice provided 53.2 ± 2.0 grams of sugar at baseline and 54.3 ± 1.9 grams at follow-up and was consumed within 10 minutes. An additional 2-4 ounces of water was used to rinse the drinking glass, and this was also consumed. The amount of $^{13}CO_2$ in the breath samples was measured by Metabolic Solutions (Nashua, NH) with a Sercon ABCA2 isotope ratio mass spectrometer (IR-MS, Sercon, Ltd., Crewe, UK). The percent atom excess in delta per million (parts per thousand) ^{13}C relative to the international standard Vienna Pee Dee Belemnite (Berglund and Weiser, 2011) was used to calculate octanoate oxidation (OctOx). OctOx (**equation 1 in the method section**) was calculated as the product of steady state CO_2 production rates and the percent $^{13}CO_2$ atom excess in breath (MW octanoate: 148.18 g/mol). As shown in the paper's **figure 1**, Respiratory gases were collected once before the test and 4 times over 150 minutes. Total expired CO_2 was calculated using the trapezoidal rule. The production rate was corrected using an estimate of the body's bicarbonate pool (van Hall, 1999) and divided by the number of labels (four) within the isotope to calculate the moles of octanoate oxidized. OctOx data were considered in the following units: the fraction of dose oxidized over 135 min, and the fraction oxidized divided by total body weight, to consider a relationship with total liver weight (Chan et al., 2006).

Standard of care: Subjects randomized to the standard of care group (n = 7) were offered a single session with the study dietitian at the start of the program where the dietitian provided weight loss education and encouraged increasing physical activity. Standard care participants met with study staff again before the follow-up visit and otherwise receive medical care as directed by their physician or clinical staff independently of the research program.

Lifestyle intervention: Subjects in the lifestyle group (n = 15) participated in weekly supervised high intensity interval training (HIIT) sessions which consisted of four consecutive, four-minute intervals at 90-95% heart rate max separated by three-minute active pauses at ~50% heart rate max three days per week. Our group (Winn et al., 2018), and others (Hallsworth et al., 2015), have demonstrated HIIT is effective at reducing liver fat and NAFLD risk. All training sessions were supervised by an exercise physiologist, completed on a treadmill, cycle ergometer, or elliptical, and intensity was monitored using heart rate monitors (Polar USA). The same subjects met with the research dietitian weekly for the first two months, biweekly for months three to five, and monthly for months six to nine (and beyond). Based on current treatment guidelines, the dietitian provided personalized nutrition education with the goal of energy restriction (~500 kcal/day deficit) over the 36-week intervention period (Chalasani et al., 2018). Emphasis was placed on decreasing sugar intake and consuming adequate protein to reduce muscle mass loss.

Supplementary Table 1. Baseline diabetic status and subject medications

**Diabetes status**	***n* (%)**
Type 2 Diabetes Mellitus	18 (72%)

**Drug type ***	***n* (%)**
Metformin	17 (68%)
Insulin	4 (16%)
Sulfonylurea	3 (12%)
Statins	12 (48%)
DPP4 inhibitor	2 (8%)
GLP-1 agonists	6 (24%)
SGLT-2 inhibitors	2 (8%)

*Baseline medication use in Part 1 of this study (n = 25)

Supplementary Figure S1. Part 2: Individual changes in NAFLD activity scores, steatosis, and total octanoate oxidized (OctOx)

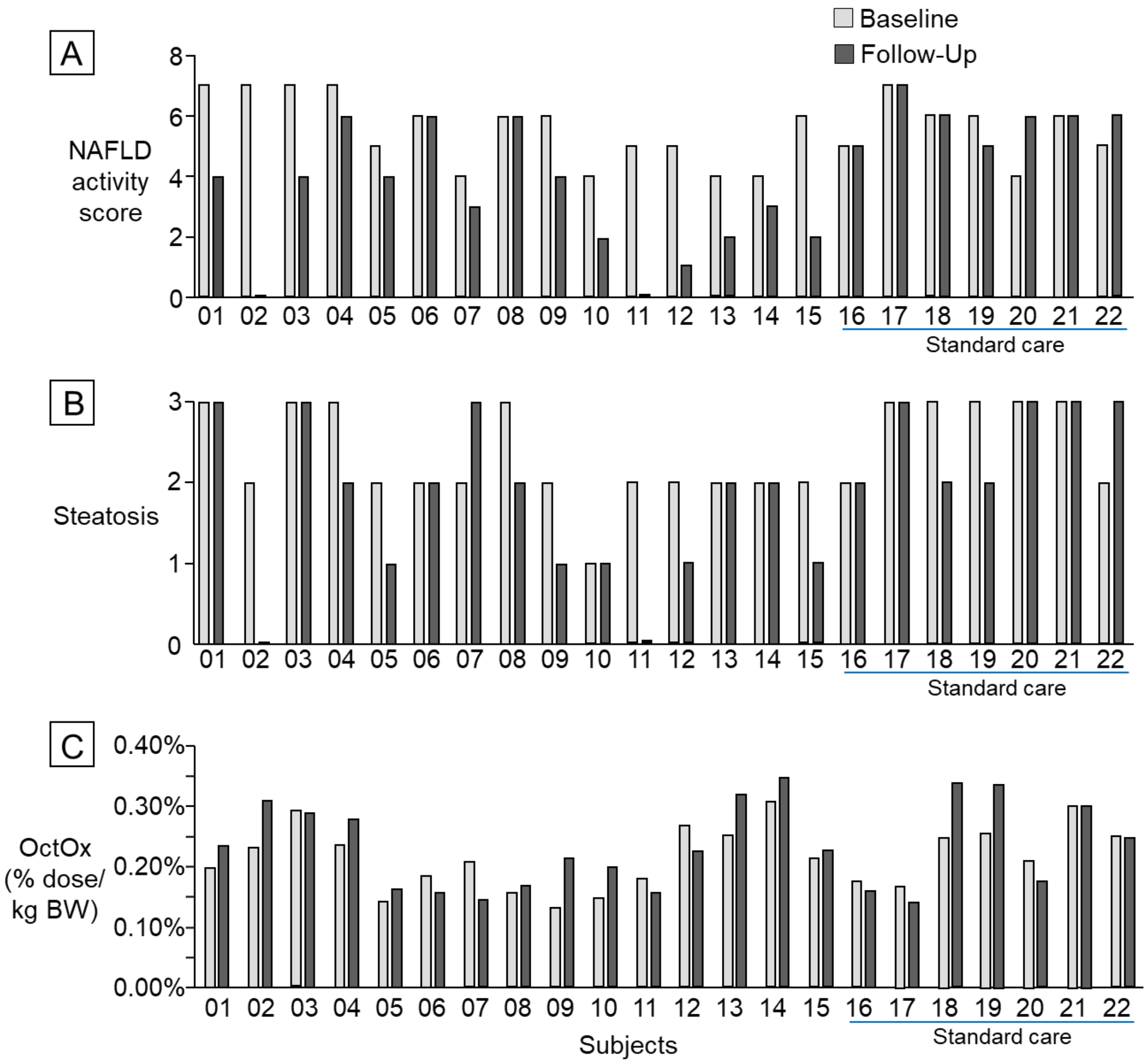

A-C: Part 2 individual subject data from baseline to follow-up for (A) NAFLD activity score, (B) steatosis, and (C) OctOx in units of the percentage of the total dose administered that was oxidized per kg body weight over 135 minutes.

Note: The participants who received lifestyle treatment were numbers 1-15. Standard of care are subject numbers 16-22. Small bars just above the x-axis indicate a score of zero (subjects 02 & 11) for NAFLD activity score and steatosis.
